# Supplementary figures and images for: Arsenic(III)-induced oxidative defense and speciation changes in a wild Trametes versicolor strain
Source: PLoS One. 2023 May 30;18(5):e0286105. doi: 10.1371/journal.pone.0286105 (PMC10228811; doi:10.1371/journal.pone.0286105)

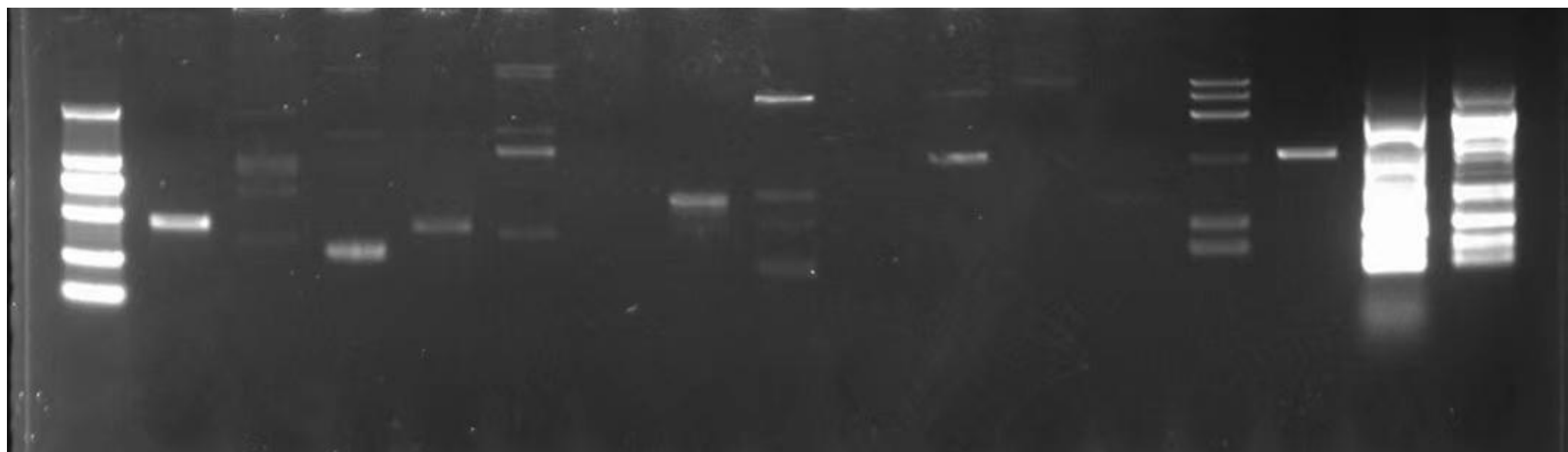

Supplement: S1 Raw images — (PDF) [file pone.0286105.s001.pdf]
