# Supplementary material for: Arsenic(III)-induced oxidative defense and speciation changes in a wild Trametes versicolor strain
Source: PLoS One. 2023 May 30;18(5):e0286105. doi: 10.1371/journal.pone.0286105 (PMC10228811; doi:10.1371/journal.pone.0286105)
Supplement: Attachment — Submitted filename: Respond to Reviewers.docx [file pone.0286105.s002.docx]

**Response to Journal Requirements**

Point 1. Please ensure that your manuscript meets PLOS ONE's style requirements, including those for file naming. The PLOS ONE style templates can be found at 
https://journals.plos.org/plosone/s/file?id=wjVg/PLOSOne_formatting_sample_main_body.pdf and 
https://journals.plos.org/plosone/s/file?id=ba62/PLOSOne_formatting_sample_title_authors_affiliations.pdf

**Response 1:** Thank you for your message. I have downloaded two PDF files and made some changes about style.

(1) According to authors affiliations requirements, we have added departments & province information, changed some symbols and deleted some unnecessary information about corresponding authors(shown in the picture below).
(2) According to style requirements about main body, we have added a blank before every paragraph, made a change from “ Fig.1” to “Fig 1”, deleted “Funding information” & “Conflict of interests”, resubmitted images files(Fig 1-8 )with TIF style and added a conclusion.

“Conclusion This study evaluated As tolerance and accumulation capacities of a wild *T. versicolor* strain under As stress. Some tolerance/detoxification mechanisms have been also generalized in this fungus. Results indicated that *T. versicolor* may be one of the few mushrooms with simultaneous As accumulation and tolerance capacities. The detoxification mechanisms of this strain were involved alleviating the toxicity by increasing the activities of antioxidants, especially GSH, as well as by converting As ^III^ into As ^V^ and other less toxic As species. These above which were not in agreement with previous results obtained from plants will require further study and confirmation. *T. versicolor* might be used as a bio-accumulator to deal with As exposure in As-contaminated environments based on its extraordinary As tolerance and accumulation capacities.” (**P11, L432-441**)

Point 2. We note that the grant information you provided in the ‘Funding Information’ and ‘Financial Disclosure’ sections do not match. When you resubmit, please ensure that you provide the correct grant numbers for the awards you received for your study in the ‘Funding Information’ section.

**Response 2:** Thank you. We have deleted “Funding information” section in the revised manuscript and made a change in the funding information.

Point 3. We note that you have stated that you will provide repository information for your data at acceptance. Should your manuscript be accepted for publication, we will hold it until you provide the relevant accession numbers or DOIs necessary to access your data. If you wish to make changes to your Data Availability statement, please describe these changes in your cover letter and we will update your Data Availability statement to reflect the information you provide.

**Response 3:**We will contact with you if we wish to make changes to the DATA AVAILABILITY STATEMENT.

Point 4. PLOS ONE now requires that authors provide the original uncropped and unadjusted images underlying all blot or gel results reported in a submission’s figures or Supporting Information files. This policy and the journal’s other requirements for blot/gel reporting and figure preparation are described in detail at https://journals.plos.org/plosone/s/figures#loc-blot-and-gel-reporting-requirements and https://journals.plos.org/plosone/s/figures#loc-preparing-figures-from-image-files. When you submit your revised manuscript, please ensure that your figures adhere fully to these guidelines and provide the original underlying images for all blot or gel data reported in your submission. See the following link for instructions on providing the original image data: https://journals.plos.org/plosone/s/figures#loc-original-images-for-blots-and-gels. 
In your cover letter, please note whether your blot/gel image data are in Supporting Information or posted at a public data repository, provide the repository URL if relevant, and provide specific details as to which raw blot/gel images, if any, are not available. Email us at plosone@plos.org if you have any questions.

**Response 4:** We have submitted a PDF file with naming “S1 Fig” in Supporting Information files section. This will provide the original underlying image for Fig 1. Furthermore, we also provide ITS Genbank No.(OL831142) of this strain to ensure the authenticity of Fig 1-2.

Point 5. PLOS requires an ORCID iD for the corresponding author in Editorial Manager on papers submitted after December 6th, 2016. Please ensure that you have an ORCID iD and that it is validated in Editorial Manager. To do this, go to ‘Update my Information’ (in the upper left-hand corner of the main menu), and click on the Fetch/Validate link next to the ORCID field. This will take you to the ORCID site and allow you to create a new iD or authenticate a pre-existing iD in Editorial Manager. Please see the following video for instructions on linking an ORCID iD to your Editorial Manager account: <https://www.youtube.com/watch?v=_xcclfuvtxQ>

**Response 5:** We have finished updating some information and authenticating a ORCID for the corresponding author.

Point 6. Please include captions for your Supporting Information files at the end of your manuscript, and update any in-text citations to match accordingly. Please see our Supporting Information guidelines for more information: http://journals.plos.org/plosone/s/supporting-information.

**Response 6:** According to guidelines for supporting information, we have resubmitted Supporting Information files, added captions for theses files at the end of the manuscript [“S1 Fig. Raw and original underlying image for Fig 1.” (**P15, L604-605**)] and update in-text citations [ “Fig 1 and Fig in S1 Fig” (**P5, L207**)]

**Response to Reviewer 1 Comments**

**Point 1 :**Title: The title is meaningless and needs a complete change.
**Response 1:** We appreciate your feedback and are glad to hear that our manuscript can be improved. We have replaced a new title in the revised manuscript: “Arsenic(III)-induced oxidative defense and speciation changes in a wild *Trametes versicolor* strain” (**P1, L1-2**)

**Point 2:** Abstract: The abstract lack a proper introduction, purpose, and methodology. These three items must be added to the .
**Response 2:** Thank you for your feedback. According to this comment point, we have focused on purpose & methodology and rewrited the Abstract section. “Oxidative defense or arsenic(As) changes exhibited by *Trametes versicolor* in response to toxicity under As stress remain unclear. In this study, after internal transcribed spacer identification, a wild *T. versicolor* HN01 strain was cultivated under 40 and 80 mg/L of As ^III^ stress. The antioxidant contents by multifunctional microplate reader and the speciations of As by high performance liquid chromatography in conjunction with inductively coupled plasma mass spectrometry were examined to explore the detoxification mechanisms. “(**P1, L15-19**)

**Point 3:** Introduction: In this section, the authors presented good info about the contamination of As. However, the impact of As contamination on human health and As toxicity in the food chain reaching ultimate consumers (animals and plants) were not mentioned in the “Introduction”.
The lines from 86 to 94 are not necessary and need summarization.
Depending on what is presented in the “Introduction”, the last paragraph should focus on the aims of the study. The aims were not clear.
**Response 3:** (1) According to this reviewer 1’s comment, we have added some comments and citations about the impact of As contamination on human health and As toxicity in the food chain. “As a metalloid, arsenic (As) is a major cause of life-threatening degenerative diseases affecting humans such as cancer, liver injury and inflammatory diseases[9-11]. It also enters the food chain and becomes a major threat to food safety[12].” (**P1, L41-43**)

(2) We have summarized the comments in lines from 86-94. “High performance liquid chromatography (HPLC) in conjunction with inductively coupled plasma mass spectrometry (ICP-MS) because of high sensitivity and a wide linear dynamic range[25] has emerged as one of the best speciation analysis techniques. HPLC ICP-MS is used in this study to examine the compositions of the As forms in *T. versicolor* and further understand the detoxification methods from the perspective of compound transformations.” (**P3, L91-95**)

(3) According to this reviewer 1’s comment, we have focused on the aim and made some changes in the last paragraph of Introduction section. “Some plants or fungi with extraordinary heavy metal tolerance have received increasing attention and trigger further study about detoxification mechanism. After a wide *T. versicolor* identified by internal transcribed spacer (ITS) and incubated under different concentration of As stress, we focused on its tolerance and accumulation capacities of As, its detoxification mechanisms based on antioxidants and speciation analysis to unlock its bio-accumulator and mycoremediation potentials.” (**P3, L96-100**)

**Point 4:** Materials and Methods:
In general, this section contains many grammar mistakes and sometimes not clear sentences.
Strain, chemicals and medium
The first paragraph should revise, “The T. versicolor HN01 strain was obtained from China Center for Type Culture Collection (CCTCC M 2021010)” without further information. The culture conditions of the strain should mention.
The origin/company of chemicals should mention.

Internal transcribed spacer (ITS) molecular identification and the phylogenetic tree
1) Indicate the forward and reverse primers used for ITS1 and ITS4.
2) Did the authors check the purity of extracted DNA?
Statistical analysis
The p value should be small and Italic.

**Response 4:** (1)About the first paragraph, because the reviewer1 has not pointed out what the further information is, we understand the culture conditions of this strain should be added. “The isolated tissue was incubated in the dark at 24°C for 10 days with potato dextrose agar (PDA) as medium.” (**P3, L106-107**). And we also have added a origin information of chemicals. “Pre-dried to achieve constant weight, 8.6699 g NaAsO_2_ (CAS7784465, Sigma-Aldrich,USA) were dissolved…”(**P3, L108**)

(2)About the primers in ITS Section, I apologize for any confusion caused. After reviewing the former manuscript, I confirm that we have indicated the forward and reverse primers for ITS1 and ITS4. “ITS1 (5 '-TCCGTAGGTGaACCTGCGG-3') and ITS4 (5 '-TCCTCCGCTTATTGATATG-3') were used as the two primers for the PCR amplification. “(**P3, L122-123**).Therefore, we cannot address this point. About the purity of DNA, we conducted purity measurements of extracted DNA and found that the value of OD260/OD280 was about 1.9. However, We apologize for omitting this information in former manuscript and added it. “and the length was approximately 527 bp (Fig 1 and Fig in S1 Fig) and the value of OD260/OD280 was about 1.9.” (**P5, L207-208**)

(3) According to the advice, we have changed the style of *p* value from “*P”* to “*p”* in Materials &Methods Section and other sections.

**Point 5:** Results
ITS Molecular identification and phylogeny of the Trametes species
Can the authors demonstrate the purpose of this experiment? The strain used in their research was already isolated, identified, and authenticated by China Center for Type Culture Collection. Is this strain isolated from contaminated area or showed significant tolerance or accumulation of metal contamination?
I think this step is irrelevant with the context or the purpose of the manuscript.
Hyphae growth 213 of T. versicolor and HIR
The figure 3 is not clear, I highly recommend the authors to replace this figure (the plates). The non-differences are quoit obvious, however neither the hypha growth nor the darkness were observed. I prefer to add PDA without growth as blank.
Replace CK group with control group whenever mentioned, including figures and tables.

As speciation in the hyphae of T. versicolor under As stress
The results are very conceived. **Response 5:** Thank you for your feedback.

(1)About the purpose of ITS experiment, we confirm this part is relevant with the purpose of the manuscript. It provided us authentic and reliable research materials. Firstly, different from identification based on the six organs in plants, the identification of fungi is only based on the morphological characteristics of the fruiting body, which is difficult to identify a species of fungi. Moreover, in the poraceae, there are some species(such as T. trogii, Stereum ostrea, etc.) with highly similar appearance and morphology to Tramtes versicolor. ITS identification can make up the shortage of identification of appearance characters in fungi. At present, ITS has been successfully applied to the identification of porcini (Zhang Bingbing et al., 2021), morchella (Zhao Ruihua et al., 2022), poromyces obliquus (Yin Yuhan et al., 2021), Ganoderma lucidum (Xing Wei et al., 2021) and other fungi strains. Secondly, this strain originated from wild. We collected, isolated, ITS identified and authenticated it, not by China Center for Type Culture Collection (CCTCC). CCTCC only helps us store this strain and give a number for storage. Thirdly, enrichment and tolerance to As about Tramtes versicolor have not been reported in any literature. Did only this strain show enrichment and tolerance to As or all Tramtes versicolor? We will ensure that this question is thoroughly discussed in all relevant sections of our future research.

(2) About Hyphae growth Section, we have submitted a clear Fig 3. The reviewer1 mentioned that he (or she) prefered to add PDA without growth as blank. In our opinion, if add another blank group of PDA without growth in Fig 3, it will be hard to beautify a figure by PS because of too large difference of background color in all groups. Moreover, we have replaced CK group with control group in the whole manuscript.

(3) About As speciation Section, I apologize for any confusion caused. We acknowledge that it is improper for the last sentence in our manuscript. We rewrite this sentence.”Considering As ^V^ was the dominant species in the hyphae with no stress or As stress in our research, it seemed that converting As ^III^ to As ^V^ might be partly connected with detoxification mechanism of *T. versicolor*.” (**P8, L275-277**)

**Point 6:** Discussion:
Accumulation and tolerance capacities in fungi
This sentence is irrelevant please remove it “As a medicinal mushroom from the family Polyporaceae, whether T. versicolor enriches As remains a secret”.
The included good info about mycoremediation, my suggestion is to summarize it in one paragraph.

Antioxidants and detoxification
“This result further confirmed the general lack of PCs in most fungi”, how can the authors justify this statement? Any possible methods to detect the percentage of PCs in T. versicolor after As treatments?

As speciation and detoxification
Please remove this sentence “The HPLC ICP-MS detection method was utilized to learn from herbal samples and rice [42, 43]”.
Line 407: The sentence “In this article, the biotransformation of Asv to AsIII, AsB and methylarsenic species” is incorrect. It should be “the biotransformation of AsIII to Asv.

Some hypotheses about As detoxification pathways in T. versicolor
There are some misleading suggestions about the pathway of As III detoxification to As V, which is the main finding in this manuscript. The authors proposed 3 different pathways.
Main Issue: There are many mistakes in describing the conversion event, As III is always biotransformed to As V. The authors should focus on this main pathway which involves the biotransformation of As III to As V accompanied by overexpression of GSH.
The other pathways were not supported by the results in this manuscript, so I highly recommend deleting them and invest bioinformatic tools to propose a clear pathway for As III detoxification. This recommendation also includes Fig. 8.
**Response 6:** (1) Accumulation and tolerance capacities in fungi
According to this comment point, we have removed this sentence “As a medicinal mushroom from the family Polyporaceae, whether T. versicolor enriches As remains a secret”. About the suggestion to summarize two paragraphs in one paragraph, in our opinion, remaining two paragraphs is also okay. There are two reasons as follows: firstly, the first paragraph focused on discussing accumulation capacity in fungi and the second one focused on tolerance capacity in fungi. Their topics are different. Secondly, if summarizing two into one, this paragraph will become too long because it covering too much characters and words.

(2)Antioxidants and detoxification
We appreciate your feedback and are glad to hear that our manuscript can be improved. In our study, we did not conduct an extra experiment to justify this statement “This result further confirmed the general lack of PCs in most fungi”, because we focused on evaluating tolerance and accumulation capacities of As, detoxification mechanisms based on antioxidants and speciation analysis in *T. versicolor* at the first time. It is practically challenging to consider other relevant factors simultaneously, which would compromise the objectives of our study. However, we acknowledge that it is improper for remaining this statement “This result further confirmed the general lack of PCs in most fungi” in our manuscript. We have removed this statement. And we will ensure that the two questions are thoroughly explored and discussed in our future research.

(3) As speciation and detoxification
We have removed the sentence “The HPLC ICP-MS detection method was utilized to learn from herbal samples and rice [42, 43]”.In addition, we have corrected this error. “In this article, the biotransformation of As ^III^ to As ^V^ , AsB and methylarsenic species…”(**P11, L405**)

(4) Some hypotheses about As detoxification pathways in T. versicolor
We appreciate your feedback and are glad to hear that our manuscript can be improved. According to these comments, we have deleted the subhead “Some hypotheses about As detoxification pathways in T. versicolor” and some description about other pathways hypotheses. Because not focus on discussing the pathways hypotheses of detoxification, it is not necessary for remaining Fig 8. We also deleted Fig 8, but we remained some necessary discussion based on the results in this manuscript. (**P11, L407-430**)

**Point 7:** Where is the conclusion?

**Response 7:** According to style requirements about main body, we have added a conclusion.

“Conclusion This study evaluated As tolerance and accumulation capacities of a wild *T. versicolor* strain under As stress. Some tolerance/detoxification mechanisms have been also generalized in this fungus. Results indicated that *T. versicolor* may be one of the few mushrooms with simultaneous As accumulation and tolerance capacities. The detoxification mechanisms of this strain were involved alleviating the toxicity by increasing the activities of antioxidants, especially GSH, as well as by converting As ^III^ into As ^V^ and other less toxic As species. These above which were not in agreement with previous results obtained from plants will require further study and confirmation. *T. versicolor* might be used as a bio-accumulator to deal with As exposure in As-contaminated environments based on its extraordinary As tolerance and accumulation capacities.” (**P11, L432-441**)

**Response to Reviewer 2 Comments**

**Points:**The paper is easy to follow by the reader. However, I may require some comments on the following issues.
i. Title: The title is good.
ii. Abstract: This section was well-written and easy to understand.
iii. Introduction: Adequate.
iv. Material and Methods: excellent
v. Results: Good
vi. Discussion: The discussion of the study is well written and cover the important topics.
vii. Conclusion: Excellent.

**Response :** Because the reviewer did not mention the specific problems that need to be modified, I am sorry that I cannot give a point-to-point reply based on the above comments.
