# Supplementary material for: Arsenic(III)-induced oxidative defense and speciation changes in a wild Trametes versicolor strain
Source: PLoS One. 2023 May 30;18(5):e0286105. doi: 10.1371/journal.pone.0286105 (PMC10228811; doi:10.1371/journal.pone.0286105)
Supplement: Attachment — Submitted filename: Respond to Reviewers.docx [file pone.0286105.s003.docx]

**Response to Journal Requirements**

Points: Please review your reference list to ensure that it is complete and correct. If you have cited papers that have been retracted, please include the rationale for doing so in the manuscript text, or remove these references and replace them with relevant current references. Any changes to the reference list should be mentioned in the rebuttal letter that accompanies your revised manuscript. If you need to cite a retracted article, indicate the article’s retracted status in the References list and also include a citation and full reference for the retraction notice.
Response : Thank you for your message. According to journal requirements, we have checked the status of the 53 cited papers and ensured no retracted papers in our manuscript. And we also have made some changes about Ref. 18 **(L499, red)** and Ref. 38 **(L559, red)** in Reference section.

**Response to Reviewer 1 Comments**

Points : Title: No comments.
Abstract: No comments
Introduction: Perfectly improved.
Materials and Methods:
Strain, chemicals and medium
First line: obtained by issue ----- I think they meant “by tissue”.
“Pre-dried to achieve constant weight” something missing with this sentence, needs rephrase.
Results
Hyphae growth 213 of T. versicolor and HIR
I'm still not convinced with Fig. 3 and the author’s response; the authors did not replace this Fig. with a better one. Replacing the Fig. is a vital issue, since it’s the ground base of the manuscript.
Discussion: No Comments.

Response : We appreciate your feedback about our resubmitted manuscript and are glad to hear that our manuscript can be improved further. According to your comments above, we have made some changes below.

(1) About Materials and Methods section, we had replaced “issue” with “tissue” **(L103)**. And we also rephrased this sentence. “After NaAsO_2_ (CAS7784465, Sigma-Aldrich, USA) been pre-dried to achieve constant weight, 8.6699 g sample was dissolved in 100 mL of deionized water…”**(L108-109)**
(2) About Results section, we have re-photographed these pictures and made a new Figure as Fig 3. Furthermore, You mentioned that you preferred to add PDA without growth as another blank in the first review comments, so we have added three PDA blank plates (Medium-As 0 mg/L, Medium-As 40 mg/L and Medium-As 80 mg/L) in new Fig 3. At the same time, we have changed the caption about Fig 3.
(3) Because you did not mention the specific comments about Title, Abstract, Introduction and Discussion sections, I am sorry that I cannot give a point-to-point reply based on the above comments.

**Response to Reviewer 2 Comments**

Points: I have reviewed the revised submitted article, which investigates the arsenic tolerance and detoxification mechanisms of the wild fungus Trametes versicolor HN01 under arsenic stress. The study is well-structured and presents valuable insights into the potential use of T. versicolor as a bio-accumulator for dealing with arsenic exposure in contaminated environments.
The methodology employed in the study is sound, and the results provide clear evidence of the fungus's ability to tolerate high levels of arsenic and effectively detoxify the metalloid. The findings on the role of antioxidants, especially glutathione, in detoxification, as well as the conversion of As III into As V and other less toxic species, are both novel and significant.
Although the results differ from previous plant studies, the authors recognize this discrepancy and suggest further research to confirm their findings. This honesty and transparency in the discussion only serve to strengthen the article's credibility.
Based on the quality of the research, the relevance of the findings, and the potential applications of T. versicolor in environmental remediation, I highly recommend accepting this article for publication. The study will undoubtedly contribute to the existing body of knowledge on the subject and spark further research in the field.
Response : Because the reviewer did not mention the specific problems that need to be modified, I am sorry that I cannot give a point-to-point reply based on the comments above.
